# Early Atherosclerosis in HIV Infected Subjects on Suppressive Antiretroviral Treatment: Role of Osteoprotegerin

**DOI:** 10.1155/2013/737083

**Published:** 2013-12-05

**Authors:** Alessandra D'Abramo, Claudia D'Agostino, Alessandra Oliva, Marco Iannetta, Gabriella D'Ettorre, Francesco Vullo, Massimo Mancone, Maria Rosa Ciardi, Claudio Maria Mastroianni, Vincenzo Vullo

**Affiliations:** Department of Public Health and Infectious Diseases, “Sapienza” University of Rome, Viale del Policlinico 155, 00161 Rome, Italy

## Abstract

Cardiovascular disease is increased in HIV-infected patients. Cytokines such as osteoprotegerin are implicated in atherosclerosis. The aim of our study was to evaluate the role of osteoprotegerin in the development and progression of atherosclerosis in HIV infected subjects on suppressive antiretroviral treatment. We enrolled 76 patients; 35 HIV infected men on suppressive Highly Active Antiretroviral Therapy with Framingham score <10%; 21 HIV negative individuals matched for age, gender, and Framingham score, and 20 subjects with Framingham score >10% as control groups. HIV positive subjects underwent echocardiography, electrocardiography, and heart multidetector computed tomography, whereas in HIV negative subjects, tomography was only performed in case of any abnormalities either in echocardiography or electrocardiography. In HIV positive patients, computed tomography showed stenosis in 51.4% of the subjects. Osteoprotegerin plasma levels were higher in HIV-infected patients than those in healthy controls but lower than in HIV negative subjects with Framingham score >10%. Higher osteoprotegerin plasma levels were found in HIV positive patients with grade I stenosis than in patients with grade II/III stenosis. In conclusion, in HIV infected subjects with Framingham score <10%, osteoprotegerin plasma concentrations are associated with atherosclerosis, in particular at the early stage of the process.

## 1. Introduction

Cardiovascular disease (CVD) is an emerging and significant cause of morbidity and mortality in HIV-infected patients [1]. HIV itself and antiretroviral drugs may contribute to the increased risk of CVD. In addition to traditional risk factors (age, smoking, dyslipidemia, and diabetes), chronic viral infection, immune activation, and inflammation play a central role in vascular damage and endothelial dysfunction [2]. Proinflammatory cytokines such as interleukin (IL)-6 and IL-1 are associated with development and progression of atherosclerosis [3–7]; furthermore, new soluble markers including osteoprotegerin (OPG) have been shown to be involved in this process. The OPG/RANK (receptor activator of NF-kappaB)/RANKL (receptor activator of NF-kappaB ligand), member of TNF superfamily, is a key regulatory system in bone remodelling by regulating development and activation of osteoclasts [8–11]. Recent studies showed the implication of the OPG/RANKL system in vascular disease, especially in vascular calcification, atherosclerosis and plaque formation [12–15]. There are few data about the relationship between OPG and cardiovascular disease in HIV infection; thus, the role of OPG is not yet well established. The aim of our study was to evaluate the role of OPG in the development and progression of atherosclerosis in HIV infected subjects on suppressive antiretroviral treatment.

## 2. Materials and Methods

### 2.1. Ethics Statement

The study protocol designed according to the Helsinki Declaration II was approved by the local ethics committee. All the patients gave written informed consent to participate.

### 2.2. Patients

We recruited 76 patients from the Department of Public Health and Infectious Disease and the Department of Cardiovascular, Respiratory, Nephrologic and Geriatric Sciences of “Sapienza” University of Rome (Italy). Thirty-five were HIV-infected men on Highly Active Antiretroviral Therapy (HAART) since 48 weeks with undetectable viremia (<37 copies/mL) and low cardiovascular diseases risk defined by a Framingham score <10%. Subjects with diagnosis of metabolic syndrome (waist circumference: men >102 cm and women >88 cm, triglycerides ≥150 mg/dL, HDL cholesterol: men <40 mg/dL and women <50 mg/dL, blood pressure: ≥130/85 mm Hg or use of medication for hypertension, and fasting glucose ≥100 mg/dL or use of medication for hyperglycemia) were excluded [16]. As control groups we enrolled 21 HIV negative individuals matched for age, gender, and Framingham score and 20 HIV negative additional subjects with Framingham score >10%. For each patient we collected medical and family history, lifestyle, smoking status, antiretroviral therapies, HIV-RNA zenith, and nadir CD4+ cell count. Current lymphocytes T CD4+ and CD8+ cell count were determined by flow cytometric analysis (MACSQuant Analyser Miltenyi Biotec, Germany) and HIV-1 RNA plasma levels were detected by a quantitative reverse polymerase chain reaction (Amplicor HIV monitor; Roche Diagnostic System, Branchburg, NJ version 1.5, l.o.d. 37 copies/mL). Serum glucose; triglycerides; and total cholesterol, high-density lipoprotein cholesterol (HDL), and low-density lipoprotein cholesterol (LDL) were measured in blood samples. Body mass index (BMI) was calculated (kg/m^2^) and recorded for each individual. Stress test echocardiography and electrocardiography (ECG) were performed in all patients. Ineligible participants were females, aged <18 years, previous virological failure, recent AIDS-defining illness, coinfected with hepatitis virus, patients with other comorbidities (diabetes mellitus, arterial hypertension, kidney disease, and hormonal dysfunction).

### 2.3. IL-6 and Osteoprotegerin Detection

IL-6 was measured with an ELISA kit (eBioscience bender MedSystem, Inc Vienna, Austria). The detection limit of assay was 3.1 pg/mL. Osteoprotegerin were measured by ELISA kits (Biomedica Gruppe, Vienna Austria). The detection limit of assay was 0.12 pmol/L.

### 2.4. Multidetector Computed Tomography (MDTC) and Coronary Angiography (CA)

**Figure 1:**
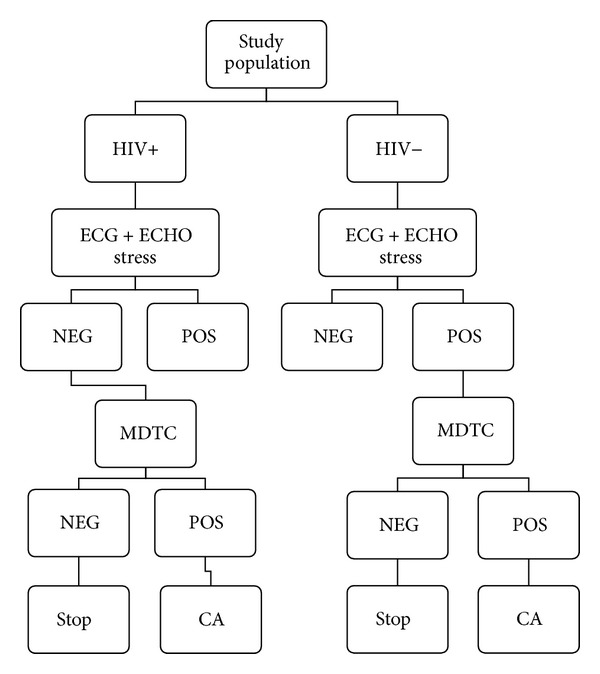
Diagnostic flowchart. Picture showing the diagnostic approach to cardiovascular disease.

The severity of CVD was evaluated in 35 HIV+ patients using a low-dose prospectively ECG-triggered CT coronary angiography protocol with 64-slice multidetector MDCT scanner (Somatom Definition Siemens medical Solution, Forehheimen, Germany) [17, 18]. MDTC dataset were transferred and analyzed through a dedicated cardiac workstation (Vitrea 2.6, Vital images, USA). For the analysis, the American Heart Association (AHA) coronary arteries segmentation model was adopted and all segments with a diameter ≥1 mm at their origin were included [19, 20]. The degree of luminal stenosis was classified as mild (grade I: 30–49%), moderate (grade II: 50–69%), severe (grade III: 70–99%), or coronary occlusion (grade IV: 100%). A threshold of 50% luminal narrowing in any coronary segment greater than 1.5 mm in diameter was adopted to define clinically significant coronary stenosis. All patients with grade II and III stenosis at MDTC were considered for coronary angiography. This was performed using standard technique with angiograms evaluated by blinded operator to the MDTC results. The decision to perform coronary revascularization was made according to the recently appropriateness criteria [21, 22]. On the other hand, HIV negative subjects underwent MDTC only in case of any abnormalities either in echocardiography or ECG; in the presence of significant stenosis a coronary angiography was set up (Figure 1).

### 2.5. Statistical Analysis

Continuous data were analyzed with Student's *t* test, whereas the nonparametric Mann-Whitney test was applied for values not normally distributed. Pearson correlation coefficient was used for correlations. Linear regression model was tested to evaluate the association between IL-6, OPG, and the grade of stenosis. Data were expressed as median and mean ± standard deviation (SD). A *P* value of <0.05 was considered statistically significant. Statistical analyses were performed using STATA (version 9) software (STATA Corp. LP, College Station, TX).

## 3. Results

### 3.1. Characteristics of the Study Population

General characteristics of the study population are summarized in Table 1. All HIV infected subjects were males with low cardiovascular diseases risk (Framingham score <10%), mean age was 53 ± 8.8 years. 57.1% were not smokers, and BMI mean was 20.7 ± 2.4 kg/m^2^. Average nadir and current lymphocytes T CD4+ cells count was 287.7 ± 253.45 cell/mmc and 570 ± 341.28 cell/mmc, respectively. The mean plasma concentrations of total cholesterol was 200 ± 51.79 mg/dL, HDL cholesterol was equal to 41.8 ± 13.67 mg/dL, LDL cholesterol was equal to 133.35 ± 47.25 mg/dL, and triglycerides were equal to 178.8 ± 90.22 mg/dL. None of the subjects was receiving lipid-lowering therapy.

### 3.2. Multidetector Computed Tomography and Coronary Angiography

MDCT examination was successfully and safely performed in all patients. We observed stenosis in 18/35 (51.4%) and no stenosis in 17/35 (48.6%). In patients with stenosis, 5/18 (27.7%) had mild stenosis (i.e., grade I) and 13/18 (72.3%) had grade II stenosis. As expected, no one had grade III stenosis. Among patients with grade II stenosis, 8/13 (61.5%) had 1 vessel and 5/13 (38.5%) 2 vessels coronary disease. All the plaques detected were eccentric and in 10/13 (76.9%) were soft. 13/13 (100%) patients with grade II stenosis at CT underwent CA and 6 (46.1%) underwent a percutaneous coronary intervention with application of 16 drug-eluting stents (Biosensors, Biomatrix DES). Twenty HIV negative subjects with CVD had grade II/III stenosis at MDTC and underwent CA; 8 (40%) underwent a percutaneous coronary intervention with application of 16 drug-eluting stents. Twenty-one HIV negative subjects with Framingham score <10% did not perform MDTC because of the absence of modifications at the echocardiography stress test and ECG.

### 3.3. Osteoprotegerin and Interleukin-6

**Figure 2:**
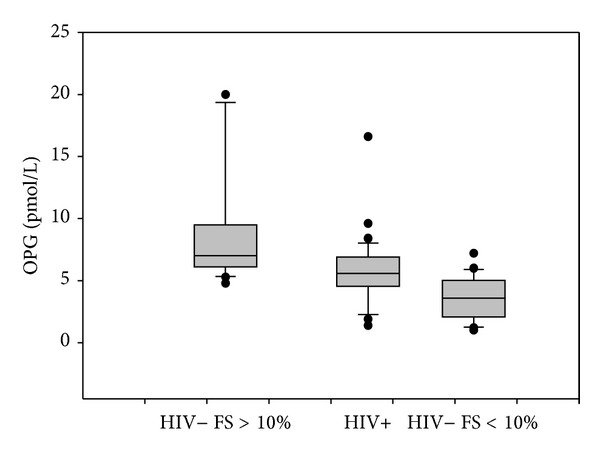
OPG plasma levels. OPG plasma levels were higher in HIV-infected patients than in healthy controls but lower than those in HIV negative subject with Framingham score (FS) >10%. Horizontal bars represent median. Upper and lower whisker mean third quartile +1.5 (Inter Quartile Range, IQR) and first quartile −1.5 (IQR).

**Figure 3:**
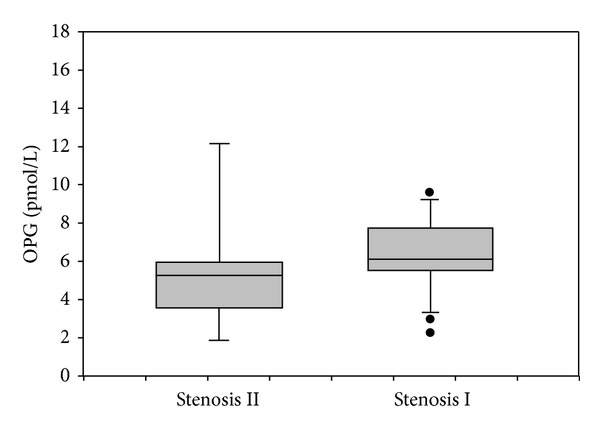
OPG and stenosis. OPG plasma levels in HIV positive patients with grade I stenosis versus patients with grade II stenosis. Horizontal bars represent median. Upper and lower whisker mean third quartile +1.5 (Inter Quartile Range, IQR) and first quartile −1.5 (IQR).

**Figure 4:**
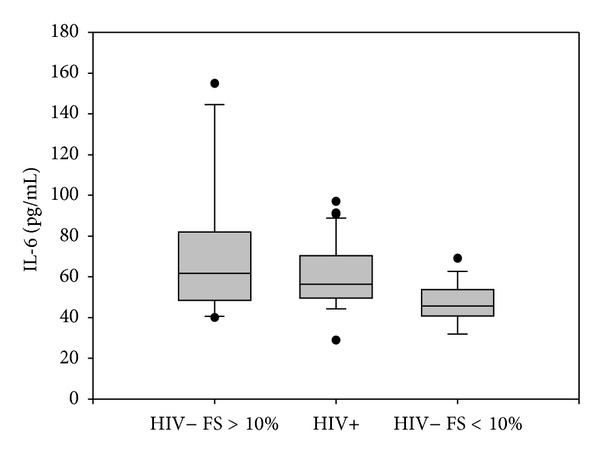
IL-6 plasma levels. IL-6 plasma levels were higher in HIV-infected patients than healthy controls but lower than those in HIV negative subject with Framingham Score (FS) >10%. Horizontal bars represent median. Upper and lower whisker mean third quartile +1.5 (Inter Quartile Range, IQR) and first quartile −1.5 (IQR).

Osteoprotegerin (OPG) plasma levels were significantly higher in HIV-infected patients than in healthy controls but lower than those in HIV negative subject with CVD (mean ± SD: 5.79 ± 2.82 versus 3.6 ± 1.7 versus 8.6 ± 4.3 pmol/L; median: 5.6 versus 3.6 versus 7 pmol/L) (*P* = 0.001) (Figure 2). We observed higher OPG plasma levels in HIV positive patients with mild coronary stenosis than in patients with grade II stenosis (mean ± SD 6.1 ± 1.5 versus 5.3 ± 1.7 pmol/L; median: 6.2 versus 5.7 pmol/L) (*P* = 0.05) (Figure 3). Interestingly, even in the 20 HIV negative subjects with CVD, OPG plasma concentration was higher in subjects with grade I stenosis than in subjects with grade II stenosis (9 ± 4.1 versus 8.1 ± 4.9 pmol/L) (*P* = 0.2). We observed negative correlation between OPG plasma levels and total cholesterol (*P* = 0.032) and LDL cholesterol (*P* = 0.089). IL-6 plasma levels were higher in HIV positive subjects than in HIV negative individuals but lower than those in HIV negative subjects with CVD (mean ± SD 60.94 ± 17.55 versus 46.78 ± 9.9 versus 72.85 ± 35.4 pg/mL; median: 58.4 versus 47 versus 61.7 pg/mL) (*P* = 0.034) (Figure 4). We observed a statistical correlation between IL-6 plasma levels and the grade of stenosis in HIV positive patients (63.8 ± 17.7 versus 51.9 ± 13.5 pg/mL) (*P* = 0.045). Moreover, in HIV negative men with CVD the IL-6 mean value was higher in men with grade II/III stenosis than in those with grade I stenosis (71.9 versus 55.1 pg/mL) (*P* = 0.244). We found a positive correlation between IL-6 plasma levels and cholesterol LDL (*P* = 0.039). We did not find any significant correlation between OPG plasma levels, IL-6 plasma levels, and immune-virological parameters.

## 4. Discussion

HIV infected adults have higher risk of cardiovascular disease than the general population due to an accelerated atherosclerosis process [23]. Several risk factors play an important role in the pathogenesis and progression of atherosclerosis in HIV patients: both HAART toxicity (mitochondrial dysfunction and oxidative stress induced by thymidine analogues or even the accumulation of abnormal laminin A associated with HIV protease inhibitor) and the virus itself induce metabolic disorders, immune activation, and chronic inflammation [24, 25].

In this study, we enrolled 35 HIV infected men on suppressed HAART by at least 48 weeks with Framingham score <10%, 21 HIV negative individuals matched for age, gender, and Framingham score and 20 subjects with Framingham score >10%. We observed an increased prevalence of coronary stenosis in HIV infected patients than in uninfected controls as suggested by the absence of any abnormalities either in echocardiography or ECG in the latter group.

Heart CT scan is an accurate instrument for noninvasive assessment of coronary arteries which can substantially contribute to the diagnosis and management of cardiovascular disease [17]. In fact, it is able to distinguish calcified, partially calcified, and noncalcified plaques to quantify stenosis, their degree, and their extension. Although heart CT is not currently recommended in asymptomatic individuals, it could be considered in patients with low cardiovascular risk because of its ability to early identify small and soft plaques.

It is known that chemokines/cytokines including IL-6 and the OPG/RANK/RANKL system are involved in endothelial dysfunction leading to atherosclerosis development and progression. IL-6 and OPG plasma levels in HIV positive subjects were higher than those in healthy controls.

IL-6 is a proinflammatory and multifunctional cytokine which is secreted by many cell types such as macrophages, T lymphocytes and endothelial cells. Several reports have consistently shown that baseline levels of IL-6 are powerful predictor of cardiovascular events [3, 4, 26]. Our data confirm a correlation between increasing IL-6 plasma levels and severity of coronary stenosis, thus suggesting the proatherogenic role of this cytokine despite HIV-RNA suppression under HAART.

As already known, there is a link between residual HIV replication, inflammation, endothelial dysfunction, and atherosclerosis; residual viral replication, persistent viral expression, and the loss of immunoregulatory cells can induce immune activation and inflammation whose persistence may result in endothelial dysfunction, vascular damage, and atherosclerosis [2].

Furthermore, OPG/RANK/RANKL system, member of TNF superfamily mostly implicated in bone remodelling, is involved in immune and in vascular system. In fact, RANKL, expressed by osteoblast cells and their precursors, activates its receptor, RANK, expressed by osteoclast cells and their precursors, promoting osteoclasts formation, activation, and prolonging osteoclasts survival. The effects of RANKL are blocked by the secretory glycoprotein osteoprotegerin which acts as a decoy receptor for RANKL. Changes in RANKL/OPG ratio are critical in the pathogenesis of bone disease [10].

The relationship between bone and vascular disease is shown; in this contest OPG could be considered as a bridge from bone to vascular system [12, 13]. The role of OPG in cardiovascular disease is still debated. In support of the antiatherosclerotic effect of OPG, recent findings showed an accelerated atherosclerotic lesion progression in OPG deficient mice [27]. Acting as a decoy receptor of RANKL, OPG blocks the RANKL effect by triggering endothelial chemotactic and angiogenic properties, by enhancing monocyte and T cell infiltration and osteogenic differentiation of multipotential vascular mesenchymal cells. On the other hand, OPG might contribute to endothelial dysfunction by blocking the RANKL signalling which is able to activate protective intracellular endothelial pathways such as the nitric oxide synthase pathway and increase the adhesion and migration of inflammatory cells through the endothelium and the activity of metalloproteases [15, 28, 29].

In our study, we found higher OPG plasma levels in HIV positive subjects than in healthy control but lower than those in HIV negative subjects with Framingham score >10% suggesting association between OPG plasma levels and cardiovascular disease. The increased OPG plasma concentration found in HIV positive patients with low cardiovascular risk may suggest that OPG is implicated in the early phase of atherosclerosis development process. Surprisingly, in HIV positive subjects we found that patients with grade I stenosis had higher OPG values rather than patients with grade II stenosis. Instead, in HIV negative patients with severe stenosis OPG plasma levels were higher than in HIV positive men. These apparently conflicting findings could be explained by the different composition and stage of the plaques; in fact, in HIV negative subjects with Framingham score >10% the calcium concentration in the plaque was higher than in HIV positive patients in whom most of the plaques were soft. OPG is expressed in different cell types including endothelial cells and vascular smooth muscle cells which are represented more in soft plaques. In this setting, OPG may indirectly prevent calcification by protecting arterial smooth muscle cells from apoptosis and OPG inactivation may lead to increased calcification which is present in older plaques [26].

Whereas in the bone system, OPG is considered as a counter-regulatory mechanism to protect bone loss; in the vascular system increased, OPG plasma levels may indicate endothelial damage [30–32]. However, further studies investigating the exact role of OPG in atherosclerosis are needed.

## 5. Conclusion

We showed that OPG plasma concentrations are associated with atherosclerosis in HIV infected subjects with a low Framingham score. OPG plasma measurement could be a useful and noninvasive tool in clinical practice in order to early discriminate subjects at risk of developing atherosclerosis.

## Figures and Tables

**Table 1:** Clinical characteristics.

	HIV− (FS < 10%)	HIV+	HIV− (FS > 10%)
Age (M ± SD)	48 ± 9	53 ± 8.8	55 ± 8
Sex	21 M, 0 F	35 M, 0 F	20 M, 0 F
Smoke status (*n*, %)			
no	15 (71%)	20 (57.1%)	6 (30%)
yes	6 (29%)	15 (42.9%)	14 (70%)
CD4+ (mmc) (M ± SD)	770 ± 221	570 ± 341.2	689 ± 197
CD4+ % (M ± SD)	36 ± 9.3	17.3 ± 7.6	33.6 ± 8.7
CD4+ (mmc) nadir (M ± SD)	—	287.7 ± 253.4	—
CD4+ % nadir (M ± SD)	—	12.9 ± 8.7	—
HIV-RNA zenith (cp/mL) (M ± SD)	—	257376.5 ± 346884.8	—
HIV-RNA (cp/mL)	<37	<37	<37
Lipid-lowering therapy			
no	21 (100%)	35 (100%)	12 (60%)
yes	0	0	8 (40%)
Systolic blood pressure (mmHg)	114.8 ± 17.5	117.3 ± 15.5	125.8 ± 18.5
Diastolic blood pressure (mmHg)	81.8 ± 11.4	77.3 ± 12.5	88.3 ± 20.5
Triglycerides (mg/dL) (M ± SD)	113.6 ± 31	178.8 ± 90.2	198.5 ± 82
Cholesterol Total (mg/dL) (M ± SD)	150.3 ± 35.8	200.7 ± 51.7	266.3 ± 48.7
Cholesterol HDL (mg/dL) (M ± SD)	53.2 ± 16.7	41.8 ± 13.6	39.8 ± 12.6
Cholesterol LDL (mg/dL) (M ± SD)	102.5 ± 32.4	133.3 ± 47.2	172.7 ± 42.4
Body mass index (kg/m^2^) (M ± SD)	18.7 ± 2.5	20.7 ± 2.4	21.7 ± 2.3
Framingham score	3.2 ± 2.1%	4.1 ± 3.2%	13.4 ± 5.4%

FS: Framingham score; M: mean; SD: standard deviation; HDL: high density lipoprotein; LDL: low density lipoprotein.
